# Sleeve-fundoplication without gastric resection vs. sleeve gastrectomy: comparable weight loss with reduced GERD incidence in obese rats

**DOI:** 10.3389/fmed.2026.1792963

**Published:** 2026-04-10

**Authors:** Minghao Zhang, Jiaqi Hu, Dalin Xu, Yun Wang, Yang Qi, Jun Bu

**Affiliations:** 1 Department of General Surgery, North Sichuan Medical College, School of Clinical Medicine, Nanchong, Sichuan, China; 2 Chengdu Second People’s Hospital, Chengdu, Sichuan, China; 3 Department of General Surgery, West China School of Medicine, Sichuan University, Sichuan University affiliated Chengdu Second People’s Hospital, Chengdu Second People’s Hospital, Chengdu, Sichuan, China

**Keywords:** bariatric surgery, gastroesophageal reflux, metabolic bariatric surgery, obesity, sleeve gastrectomy

## Abstract

**Introduction:**

Sleeve gastrectomy (SG) is a first-line bariatric procedure but may exacerbate gastroesophageal reflux disease (GERD) symptoms in obese patients. To mitigate this critical limitation, we developed a novel sleeve-fundoplication without gastric resection (SG-F) and investigated its efficacy and safety in a rat model of diet-induced obesity.

**Methods:**

A randomized controlled trial was conducted in twenty-four 8-week-old male SD obese rats, randomly assigned to the SG group (*n* = 12) or the SG-F group (*n* = 12). Postoperative complications (mortality, bleeding, GERD) and body weight changes were monitored for 6 weeks. The primary endpoint was the 6-week body weight change trajectory, and the secondary endpoint was postoperative GERD incidence.

**Results:**

No statistically significant differences were observed in postoperative mortality or bleeding rates between the two groups. The incidence of postoperative GERD was significantly lower in the SG-F group than in the SG group (0% vs. 41.7%; *P* < 0.05; RR: 0.1, 95% CI: 0.01–0.79). A significant between-group difference in body weight was noted at 4 weeks postoperatively (SG-F: 473.4 ± 18.5 g vs. SG: 513.6 ± 21.3 g; *P* < 0.05; MD: −40.2 g, 95% CI: −65.1 to −15.3 g), while the overall 6-week weight loss effect was comparable between the two procedures (*P* = 0.307).

**Conclusion:**

SG-F achieves weight loss efficacy comparable to conventional SG without gastric resection, while suggesting improved anti-reflux protection and maintaining similar safety profiles in terms of mortality and bleeding. This novel procedure represents a promising alternative bariatric option for obese patients with GERD, avoiding the need for gastric tissue excision.

## Highlights

1. Sleeve-fundoplication without gastric resection is a new bariatric procedure that reshapes the stomach without cutting any of it out and adds a Nissen wrap to curb reflux.
2. Obese rats lost a similar amount of weight with SG-F as with standard sleeve gastrectomy even though their stomachs stayed intact.
3. Zero rats in the SG-F group developed GERD, compared with 42% in the sleeve group.
4. Sleeve-fundoplication without gastric resection caused no gastric-leak deaths, making it safer than traditional sleeve gastrectomy in this study.
5. By avoiding resection yet controlling reflux, SG-F could offer obese patients with GERD a less-invasive alternative to bypass surgery.

## Introduction

Obesity is a chronic, relapsing metabolic disease characterized by excessive or ectopic fat deposition, internationally defined as a Body Mass Index (BMI) ≥ 30 kg/m^2^ (1). The global burden of obesity continues to escalate: the prevalence is projected to rise from 14% in 2020 to 24% by 2035, affecting nearly 2 billion individuals of all ages (2), and the total number of overweight and obese adults is forecasted to reach 3.8 billion by 2050, accounting for more than half of the global adult population (3). Concurrently, epidemiological studies have established high BMI as a risk factor for a spectrum of chronic diseases. Common obesity-related comorbidities include type 2 diabetes mellitus (T2DM), cardiovascular disease, obstructive sleep apnea (OSA), an increased risk of several cancers, elevated mortality, and reduced quality of life (QOL) (4).

Metabolic Bariatric Surgery (MBS) is currently the most effective treatment for obesity (5). Data from the 8th edition of the International Federation for the Surgery of Obesity and Metabolic Disorders (IFSO) Global Registry in 2022 indicate that laparoscopic Roux-en-Y gastric bypass (LRYGB) and laparoscopic sleeve gastrectomy (LSG) are the two most important metabolic bariatric procedures, with LSG being the most frequently performed MBS worldwide (6). Although LSG offers significant weight loss, relatively low technical difficulty, a low incidence of nutritional deficiencies, and marked improvement in obesity-related comorbidities, it may induce or exacerbate symptoms of gastroesophageal reflux disease (GERD) (7). Notably, obesity itself is a major risk factor for GERD, with risk increasing in parallel with rising BMI (7). Thus, the potential of LSG to induce or exacerbate GERD symptoms creates a critical clinical dilemma, as it may worsen a pre-existing comorbidity in the obese population.

Consequently, Studies have shown that Nissen fundoplication is generally considered safe and effective, significantly alleviating GERD symptoms and improving quality of life in most patients (8, 9). Therefore, some scholars have proposed combining SG with Nissen fundoplication to reduce the incidence of postoperative GERD. While laparoscopic Nissen sleeve gastrectomy (LNS) has been reported as a safe and effective bariatric approach, with a significantly lower GERD incidence compared to SG alone (10), it still requires gastric tissue excision, which remains a barrier for some patients. Some obese patients refuse metabolic bariatric surgery due to concerns about partial gastrectomy (11). To address this issue, our research team innovatively proposed a new bariatric procedure: a novel sleeve-fundoplication without gastric resection (SG-F). This study aims to investigate whether this model can provide effective weight loss while reducing GERD complications.

This study applies SG-F and conventional SG to 8-week-old obese male Sprague-Dawley rats, comparing postoperative weight loss effects and the incidence of GERD between the two groups. The objective is to determine the safety, efficacy, and feasibility of this novel approach, providing a basis for its further development to benefit patients through better weight loss outcomes, fewer complications, and improved treatment results and satisfaction.

## Materials and methods

### Experimental animals and grouping

Twenty-four 8-week-old male Sprague-Dawley (SD) rats with diet-induced obesity [specific pathogen-free (SPF) grade], with an average weight of (384.9 ± 28.2 g) were used. The rats were supplied by Beijing Vital River Laboratory Animal Technology Co., Ltd. (License No. SCXK [Jing] 2021-0006). Obesity modeling: 3-week-old SPF male SD rats were acclimatized with standard chow for 1 week, followed by feeding with a 45% high-fat diet for 4 weeks. Rats whose body weight exceeded the average weight of rats fed standard chow by 20% were defined as diet-induced obese rats (12). The obese rats were randomly assigned to the SG group (control, *n* = 12) and SG-F group (experimental, *n* = 12) using a computer-generated random number table. Sample size calculation: Based on the primary endpoint of absolute weight loss, the required sample size was five rats per group [formula: *n* = (Z_α_ + Z_β_)^2^ × 2σ^2^/δ^2^, where σ^2^, variance; δ, expected between-group difference]. For the secondary endpoint of GERD incidence, the required sample size was 10 rats per group [formula: *n* = 2pq (Z_α_ + Z_β_)^2^/(p_1_–p_2_)]. A 20% attrition rate was accounted for to account for potential mortality, with a statistical power of 90% and a two-sided α of 0.05, resulting in a final sample size of 12 rats per group. All animal experiments were approved by the Institutional Animal Care and Use Committee of the Experimental Animal Institute of Sichuan Academy of Medical Sciences and Sichuan Provincial People’s Hospital (License No. SYXK [Chuan] 2023-0058). Rats were euthanized by cervical dislocation at 6 weeks postoperatively in accordance with IACUC ethical guidelines.

### Experimental methods

#### Preoperative preparation and anesthesia

Animals were weighed, fasted for 12 h, and received an intramuscular injection of cefoperazone sodium sulbactam sodium (1.0 g, 20 mg/kg) 30 min preoperatively. Anesthesia was induced via a facemask connected to a respirator using 3% isoflurane and maintained with 2% isoflurane in 100% O_2_ at 1 L/min during surgery (13, 14).

##### SG

**FIGURE 1:**
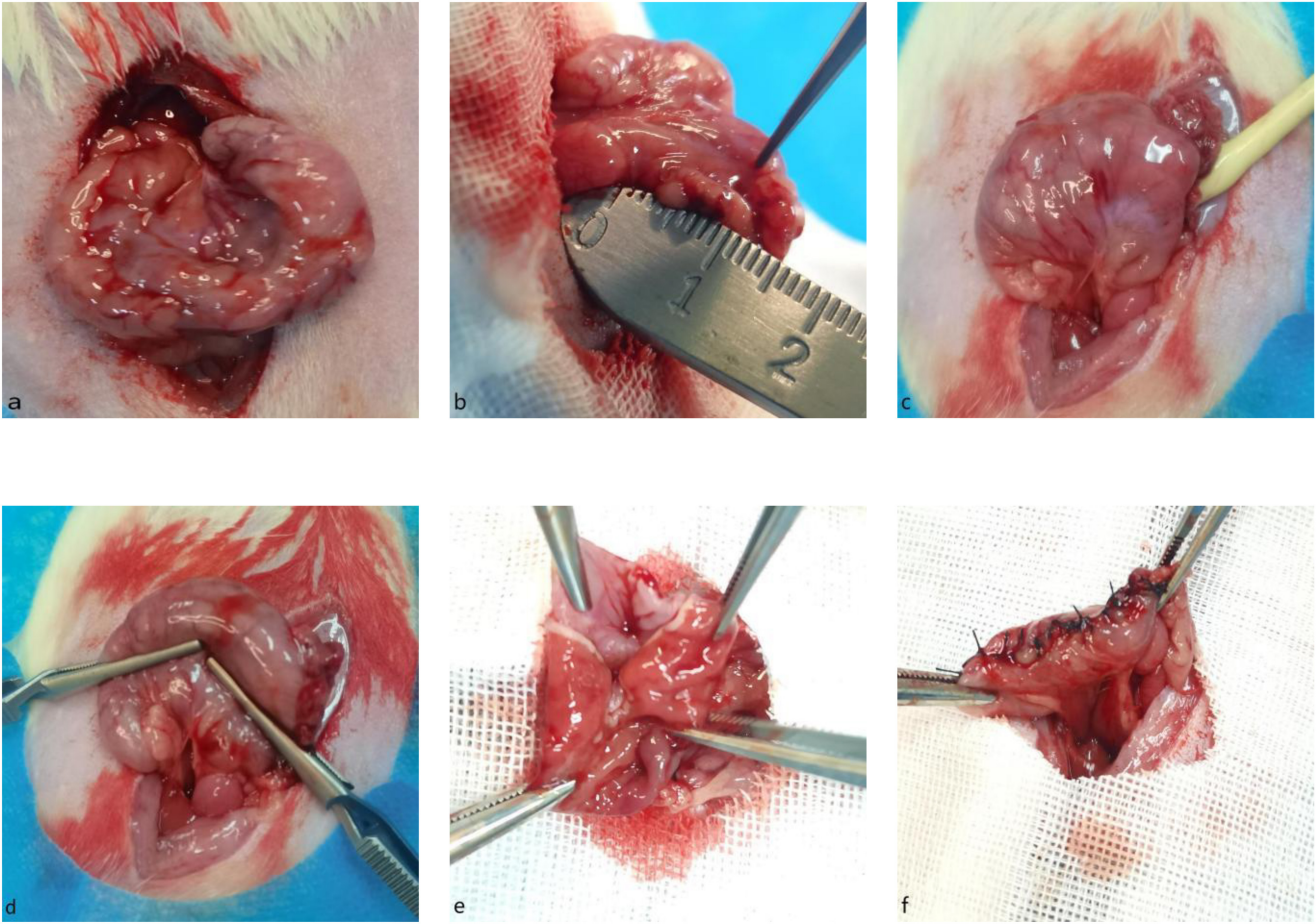
Surgical procedure of sleeve gastrectomy (SG) in a rat model. **(a)** Mobilized stomach. **(b)** Measurement of pyloric distance (5 mm). **(c)** Foley catheter positioning for shaping. **(d)** Resection line demarcation. **(e)** Stomach after incision. **(f)** Completed SG.

1. The rat was placed in a supine position and fixed on the operating table. Abdominal hair was shaved, the skin was disinfected with povidone-iodine, and sterile drapes were applied.
2. A 4.0 cm midline abdominal incision was made. The stomach was carefully exteriorized and protected with gauze moistened with saline. The hepatogastric ligament and gastrosplenic ligament were dissected. The short gastric vessels toward the splenic hilum and the gastroepiploic vessels near the pyloric antrum were ligated with 6-0 silk suture and divided (Figure 1a).
3. A transverse incision of approximately 3.0 mm was made on the anterior gastric wall along the greater curvature, about 5 mm from the pylorus (Figure 1b).
4. Through this incision, the balloon end of an 8Fr pediatric Foley catheter was inserted into the gastric lumen and advanced along the lesser curvature toward the lower esophagus for about 5 mm (Figure 1c).
5. The first clamp was applied to clamp the gastric wall directly beneath and along the catheter. Subsequently, the catheter was removed. A second clamp was placed opposite the first clamp, 5 mm from the pylorus (Figure 1d).
6. The lower edges of the two clamps defined the resection line. The gastric tissue below the clamp line was excised (Figure 1e).
7. The cut edge of the remnant stomach was disinfected with a cotton swab and closed with interrupted full-thickness sutures using 5-0 silk suture. Hemostasis was ensured, and suture line integrity was checked (Figure 1f). The stomach was returned to its anatomical position. The peritoneum, muscle layer, and skin were closed intermittently with 3-0 silk suture. Sleeve gastrectomy was completed (Figures 1a–f) (12).

##### SG-F

**FIGURE 2:**
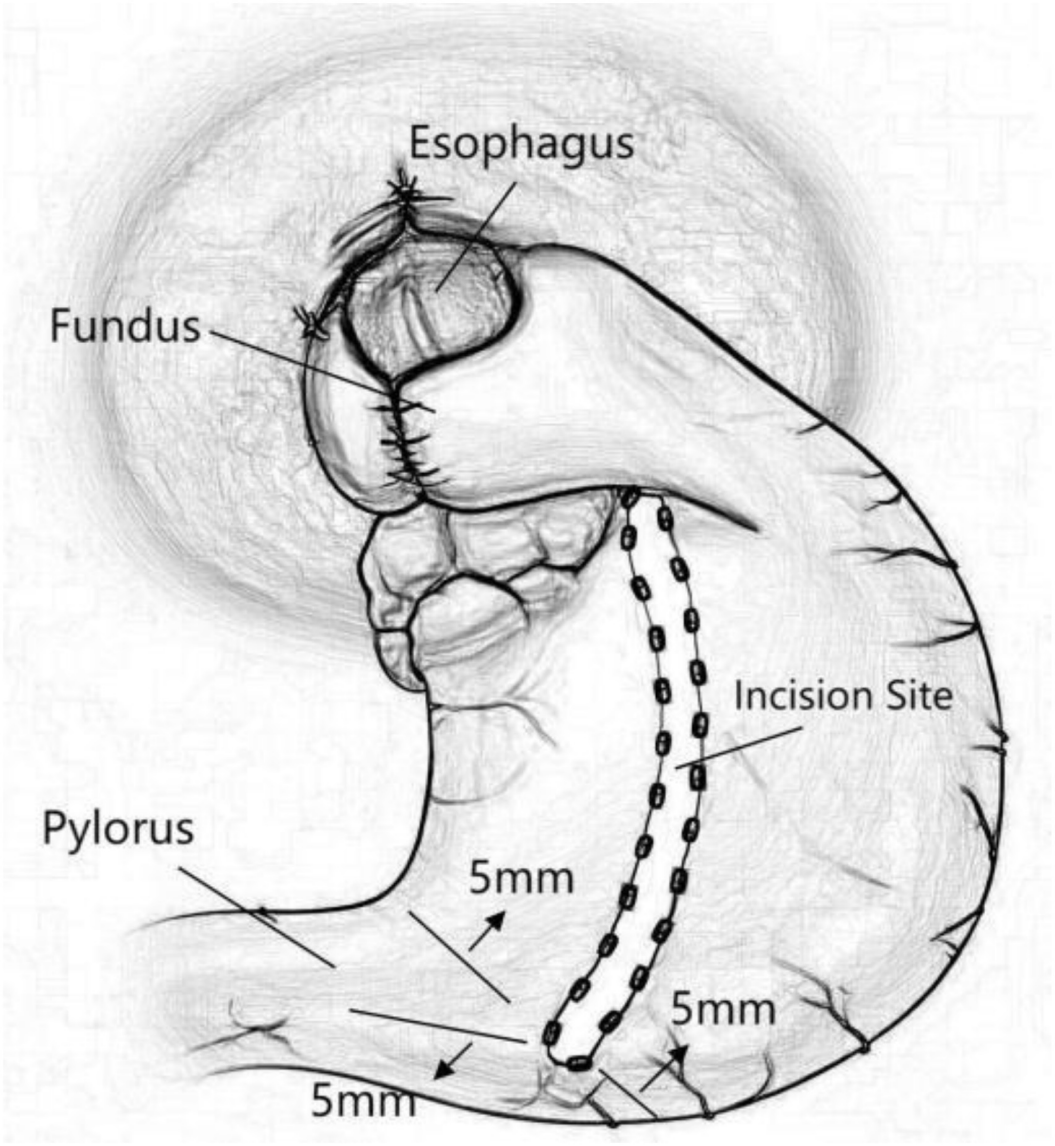
Hand-drawn schematic diagram of sleeve-fundoplication without gastric resection (SG-F).

Hand-drawn schematic diagram of SG-F (Figure 2).

**FIGURE 3:**
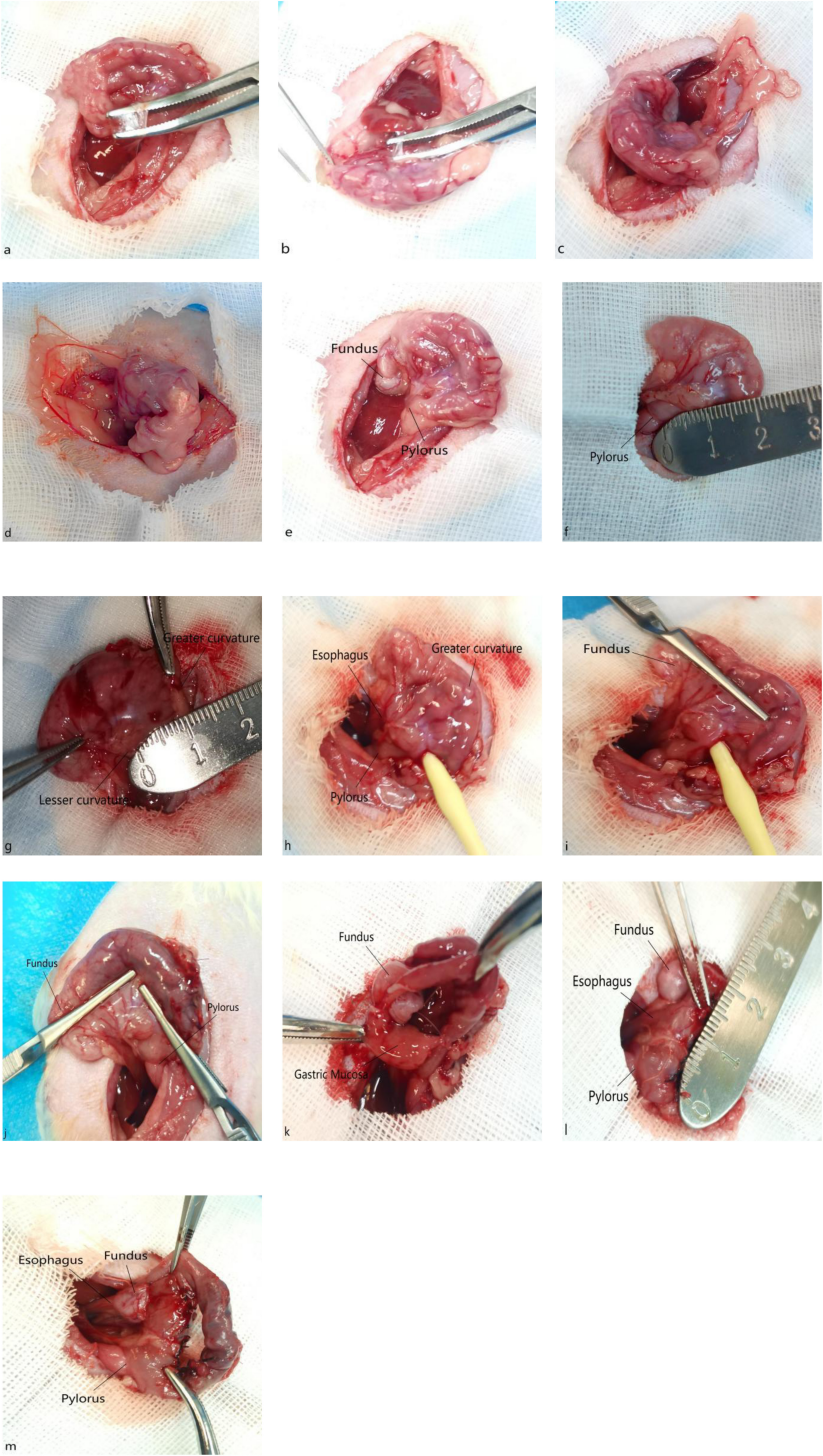
Surgical procedure of sleeve-fundoplication without gastric resection (SG-F) in a rat model. **(a)** The hepatogastric ligament. **(b)** The gastrosplenic ligament. **(c)** The short gastric vessels. **(d)** The gastroepiploic vessels. **(e)** The stomach after mobilization. **(f)** The measurement of the resection margin, approximately 5 mm from the pylorus. **(g)** The resection margins, approximately 5 mm from the greater and lesser curvatures, respectively. **(h)** The insertion of the Foley catheter for gastric shaping. **(i)** The placement of the first vascular clamp along the superior edge of the catheter. **(j)** Demonstrates the established gastric resection line. **(k)** The stomach after the incision was made. **(l)** Shows the measurement of the sutured gastric incision length, approximately 1.5 cm. **(m)** The completed SG-F procedure.

The abdomen was opened, and the stomach was mobilized following the SG steps (Figures 3a–e).
A longitudinal incision of approximately 3 mm was made 5 mm proximal to the pylorus (Figure 3f) and 5 mm from the edges of both the lesser and greater curvatures (Figure 3g).
  Through this incision, the balloon end of an 8Fr pediatric Foley catheter was inserted into the gastric lumen (Figure 3h).
The catheter was advanced along the lesser curvature toward the esophagus for about 5 mm. A clamp was then placed directly on the gastric wall beneath the catheter (Figure 3i).
After removing the catheter, a second clamp was placed parallel to the first, on the opposite side of the longitudinal incision. The lower edges of these two clamps determined the gastric resection line (Figure 3j).
The gastric tissue was incised along this line, extending to approximately 1.5 cm from the gastric fundus (Figure 3k). The gastric margins were cleaned with a cotton swab. The gastric incision was closed with interrupted full-thickness hand-sewn sutures using 5-0 silk suture (Figure 3l).
6. Subsequently, the gastric fundus was mobilized posterior to the cardiac orifice and retracted to the right side of the esophagus to complete a 360° Nissen fundoplication (esophagogastric fundal wrap). The right and left portions of the fundus were sutured together and to the anterior esophageal wall using 5-0 silk suture, completing the Nissen fundoplication (Figure 3m). Careful hemostasis was achieved, and suture line integrity was confirmed. The stomach was returned to its anatomical position. The peritoneum, muscle layer, and skin were closed intermittently with 3-0 silk suture. SG-F was completed (Figures 3a–m).

##### Postoperative care

To prevent dehydration, a subcutaneous injection of 20 ml/kg of a balanced solution (0.9% NaCl + 5% glucose) was administered immediately after surgery. For the first two postoperative days, rats received the balanced solution via oral gavage, and *ad libitum* access to standard chow was initiated on postoperative day 3 (13, 14).

##### Pathological methods

Tissue samples were fixed in 4% paraformaldehyde. After confirming adequate fixation, samples were processed according to standard operating procedures for pathological examination, including trimming, dehydration, embedding, sectioning, Hematoxylin and Eosin (H&E) staining, and mounting. Finally, pathological slides from rats in both groups were randomly assigned to a pathologist, who was blinded to the group allocation, for microscopic examination.

### Observation indicators

Rats were observed for 6 weeks postoperatively. Body weight was measured every 3 days for the first 3 weeks, and then weekly for the subsequent 3 weeks until 6 weeks post-surgery. At the 6th postoperative week, rats were euthanized by cervical dislocation according to ethical guidelines, and tissue from the lower esophagus was collected for histological examination (15–17). According to GERD pathological diagnostic guidelines, the following parameters were assessed: integrity of the lower esophageal mucosa, degree of papillary hyperplasia, thickness of the basal cell layer, and inflammatory cell infiltration. This study’s predefined pathological criteria for clinical GERD, which required marked basal cell thickening, papillary hyperplasia, and extensive inflammatory cell infiltration.

### Statistical analysis

Data analysis was performed using SPSS Statistics software (version 27.0). Continuous data are presented as mean ± standard deviation (SD), and categorical data as counts and percentages. Normality of continuous data was assessed using the Shapiro-Wilk test, and homogeneity of variances using Levene’s test. Between-group comparisons of body weight were performed using independent samples *t*-test (normal distribution, equal variance), Mann-Whitney U test (non-normal distribution), or repeated measures analysis of variance (ANOVA) for longitudinal weight changes. Comparisons of categorical complications (mortality, bleeding, GERD) between groups were performed using Fisher’s exact test. A two-sided *P* < 0.05 was considered statistically significant, and 95% confidence intervals (CI) were reported. The incidence of postoperative GERD was analyzed using an intention-to-treat (ITT) approach.

## Results

### Postoperative complications (mortality, bleeding, GERD)

Two rats died postoperatively in both the control and experimental groups. In the control group (SG), one rat died on postoperative day 1 due to intragastric bleeding, and one died on day 6 due to gastric leak. In the experimental group (SG-F), one rat died on postoperative day 1 due to intra-abdominal bleeding, and one died on day 6 due to gastric leak. There was no statistically significant difference in mortality between the two groups.

Additionally, in the control group, four rats experienced postoperative bleeding; in the experimental group, two rats experienced postoperative bleeding. However, there was no statistically significant difference in bleeding rates between the two groups. At 6 weeks postoperatively, five rats in the SG group (41.7%) exhibited GERD-like histological changes, whereas no rats in the SG-F group showed such changes (0%), with a statistically significant between-group difference (*P* = 0.037, Table 1).

**TABLE 1:** Postoperative complications in two rat groups.

Complication	SG (*N* = 12)	SG-F (*N* = 12)	*P*-value
Mortality	2	2	1
Gastric leak	1	1	1
Bleeding	4	2	0.640
GERD	5	0	**0.037**

In statistics, decimal numbers are rounded to the nearest whole number; bold *P*-values indicate statistically significant differences (*P* < 0.05).

### Body weight

**FIGURE 4:**
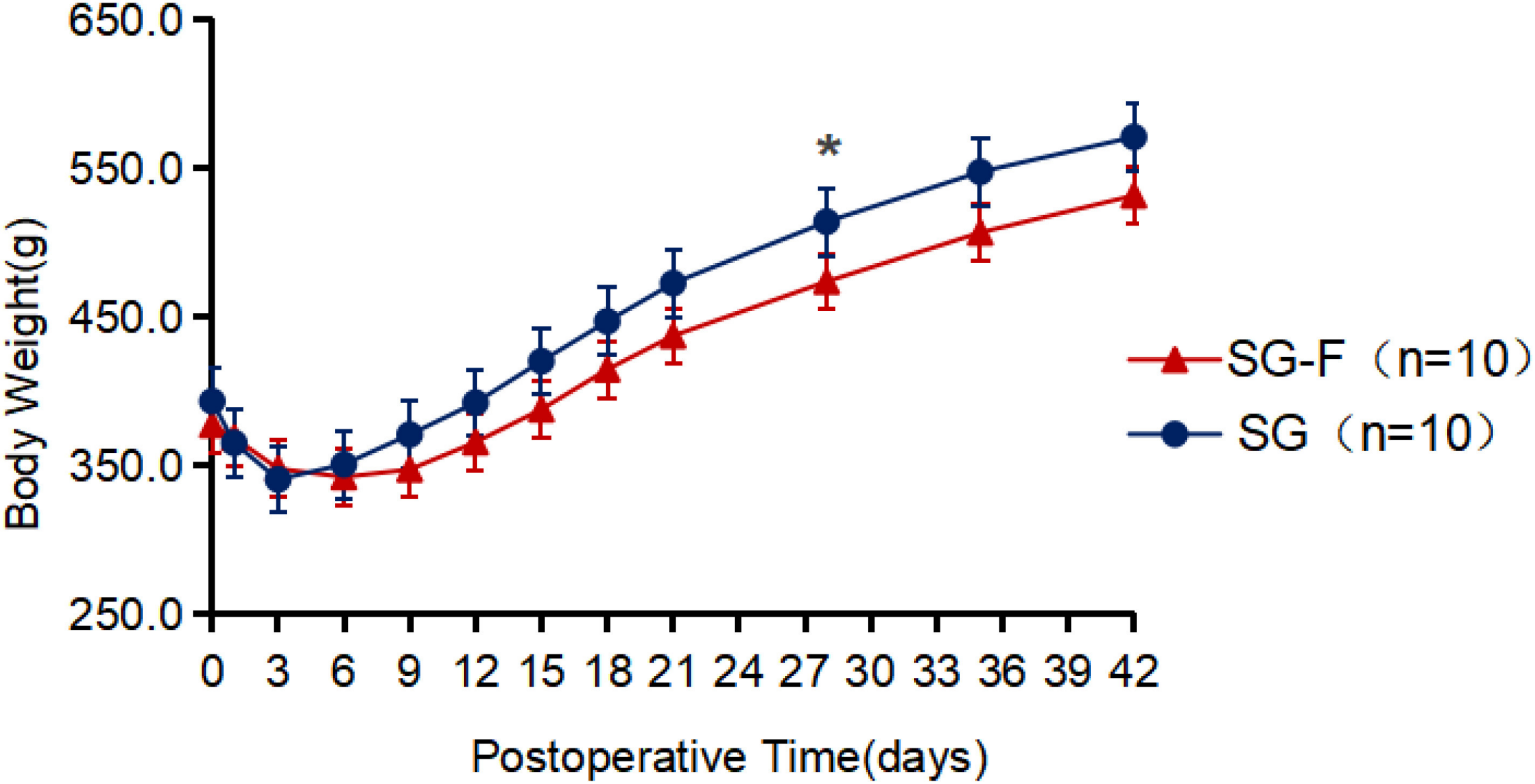
Preoperative and postoperative body weight change curves for rats in the sleeve gastrectomy (SG) and sleeve-fundoplication without gastric resection (SG-F) groups. As weight loss outcomes could not be assessed in rats that died postoperatively, these rats were excluded from this comparison, leaving 10 rats per group. SG denotes traditional sleeve gastrectomy (*n* = 10); SG-F denotes sleeve-fundoplication without gastric resection (*n* = 10). Body weight differed significantly between the two groups on POD 28. Error bars represent standard error of the mean, *P < 0.05.

Body weight analysis was limited to surviving rats (10 rats per group), as deceased rats could not be assessed for weight loss. No significant difference in preoperative body weight was observed between the two groups (*P* > 0.05). Postoperatively, body weight decreased gradually in both groups: the SG group reached maximum weight loss on POD3, with weight recovery starting on POD4 and returning to near-preoperative levels by POD13. The SG-F group reached maximum weight loss on POD6, with weight recovery starting on POD7 and returning to near-preoperative levels by POD15. Throughout the 6-week observation period, SG-F group rats had consistently lower body weight than SG group rats, with a statistically significant difference at 4 weeks postoperatively (SG-F: 473.4 ± 18.5 g vs. SG: 513.6 ± 21.3 g; *P* < 0.05; MD: −40.2 g, 95% CI: −65.1 to −15.3 g; Figure 4). However, the overall 6-week absolute weight loss was comparable between the two groups (*P* = 0.307).

### GERD

**FIGURE 5:**
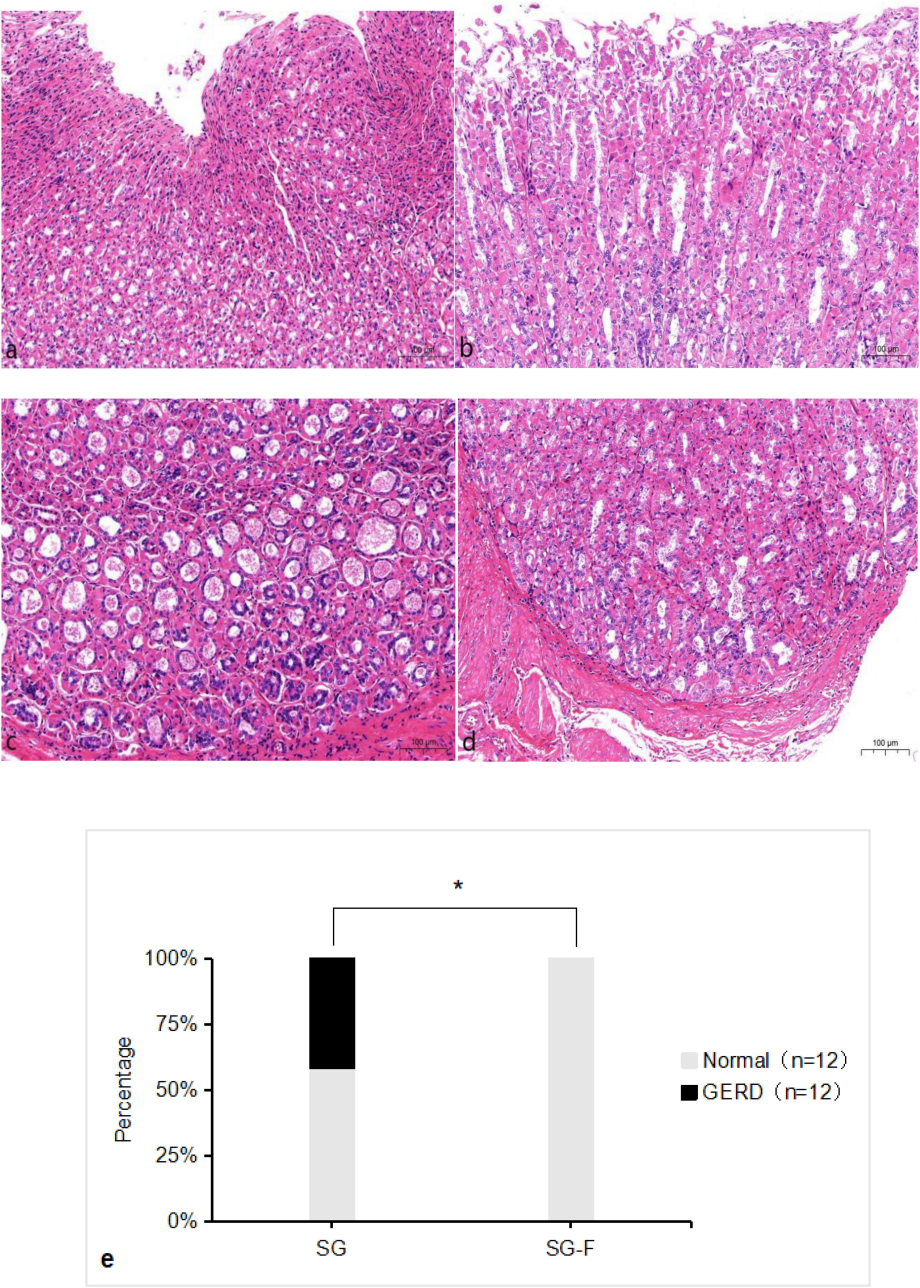
Histopathological findings. **(a)** Hematoxylin and Eosin (H&E)-stained section of a lesioned esophagus from a rat in the sleeve-fundoplication without gastric resection (SG-F) group. Shows mild inflammatory cell infiltration, moderate thickening of the basal cell layer, and no papillary hyperplasia. **(b)** H&E-stained section of a normal esophagus from a rat in the SG-F group. **(c)** H&E-stained section of a lesioned esophagus from a rat in the SG group. Shows extensive inflammatory cell infiltration, marked thickening of the basal cell layer, and mild papillary hyperplasia. **(d)** H&E-stained section of a normal esophagus from a rat in the SG group. **(e)** Incidence of gastroesophageal reflux disease (GERD) in rats of the SG and SG-F groups at 6 weeks post-surgery, *P < 0.05. The incidence of postoperative GERD in the two groups was analyzed using the ITT method, hence 12 rats per group.

According to the ITT analysis, H&E staining of esophageal tissues at 6 weeks post-surgery revealed a significantly higher proportion of rats with GERD-like histological changes in the SG group than in the SG-F group (5/12 vs. 0/12, *P* < 0.05; RR: 0.1, 95% CI: 0.01–0.79) (Figure 5e). One rat in the SG-F group presented mild histological features suggestive of reflux, including mild inflammatory cell infiltration and moderate basal cell layer thickening without papillary hyperplasia (Figures 5a–d). However, this finding did not meet the study’s predefined pathological criteria for clinical GERD, which required marked basal cell thickening, papillary hyperplasia, and extensive inflammatory cell infiltration. Thus, the SG-F group was categorized as having a 0% GERD incidence for ITT analysis.

## Discussion

This study developed and validated SG-F, a novel sleeve-fundoplication without gastric resection that overcomes key limitations of conventional SG and LNS (18). It preserves gastric integrity by avoiding irreversible resection and provides robust anti-reflux protection via 360° Nissen fundoplication. In diet-induced obese rats, SG-F achieved weight loss comparable to SG, with markedly lower postoperative GERD incidence and comparable mortality and bleeding rates, demonstrating its potential as a clinical alternative for obese patients with GERD. Amid the rising global obesity burden, innovative bariatric surgeries optimizing outcomes and reducing complications are urgently needed (1, 3), and this preclinical study confirms SG-F fulfills this need by combining effective weight loss and anti-reflux efficacy with preserved gastric anatomy, validating its feasibility, safety and efficacy in obese rats.

This study, the observed weight loss trajectories indicated that the SG-F procedure yielded favorable outcomes. Notably, the SG-F technique does not involve gastric resection and preserves the native anatomy. While the LNS procedure has been reported as a safe and effective bariatric approach, with a significantly lower GERD incidence compared to SG alone (10), it still necessitates the removal of gastric tissue. Some patients with obesity express concerns about, or decline, weight-loss surgeries that require partial gastrectomy (9). The SG-F technique could potentially alleviate such apprehensions, thereby encouraging this patient population to opt for surgical intervention. Consequently, by avoiding gastric resection, the SG-F procedure may achieve weight loss outcomes comparable to those of standard SG. In addition, SG is known to reduce gastric volume and modulate key gut hormones including ghrelin, peptide YY (PYY), and glucagon-like peptide-1 (GLP-1) (5). Studies by Wang et al. showed decreased ghrelin and increased GLP-1 and PYY levels after SG (19), and these hormones play integral roles in post-SG weight loss (20–22). Although SG-F does not involve gastric resection, it functionally partitions the stomach to reduce effective gastric volume and food contact surface area—an effect that likely mimics the gut hormone modulation observed after SG [decreased ghrelin, increased GLP-1 and PYY (19, 20)]. This partitioning may also slow gastric emptying [via altered ghrelin/GLP-1 balance (23, 24)], contributing to the sustained weight loss observed in the SG-F group at 4 weeks postoperatively. These findings provide a novel theoretical framework for bariatric surgery: weight loss can be achieved via functional gastric modification rather than irreversible resection, with gut hormone modulation as a key underlying mechanism. Future studies will validate this hypothesis via serum hormone assays and gastric emptying rate measurements.

The most compelling histopathological finding emerged from the assessment of esophageal tissues. Although one rat in the SG-F group presented mild histological features suggestive of reflux, including mild inflammatory cell infiltration and moderate basal cell layer thickening without papillary hyperplasia. However, this finding did not meet the study’s predefined pathological criteria for clinical GERD, which required marked basal cell thickening, papillary hyperplasia, and extensive inflammatory cell infiltration. Thus, the SG-F group was categorized as having a 0% GERD incidence for ITT analysis. Additionally, the 2 deceased rats in the control group were defined as deaths due to GERD onset. Therefore, the incidence of GERD at 6 weeks postoperatively was 0 case in the experimental group and five cases in the control group. This study, the incidence of GERD in the control group was 41.7%. Whereas a meta-analysis by Trujillo et al. encompassing 22 studies with 20,495 participants reported a post-LSG GERD rate of 58% (25). Therefore, the GERD incidence observed in our control group falls within the expected range reported in the literature. Importantly, the experimental group demonstrated significantly less inflammation, basal cell hyperplasia, and papillary hyperplasia—hallmark features of reflux esophagitis—with a statistically significant difference in reflux-related outcomes between the two surgical approaches. These findings provide robust morphological evidence supporting reduced GERD incidence of the combined procedure. This finding is crucial in the context of recent meta-analyses, such as that by Principe et al. which clearly showed that adding Nissen fundoplication to SG significantly reduces the risk of postoperative GERD (8). Furthermore, Savvala et al. through 5-year follow-up, found significant advantages of SG combined with Nissen fundoplication in improving postoperative GERD (18). Moreover, The study by Tadé et al. demonstrated that, for patients with primary GERD, Nissen fundoplication provides superior anti-reflux efficacy compared to other types of fundoplication procedures (26). This study builds upon this by demonstrating that the protective effect of Nissen fundoplication against GERD can be achieved without gastric resection, potentially mitigating other long-term risks of SG, such as nutritional deficiencies. Additionally, by incorporating Nissen fundoplication, this technique may be suitable for patients with obesity with pre-existing GERD, who are often recommended Roux-en-Y gastric bypass (RYGB) (27). However, RYGB alters normal anatomy significantly. This novel procedure offers a less invasive yet potentially effective alternative.

This study has several notable limitations that warrant consideration. First, the study was conducted in young male Sprague-Dawley rats, and results cannot be directly extrapolated to humans—rodent models do not fully replicate human GERD pathophysiology or the long-term metabolic effects of bariatric surgery (28, 29). Additionally, only male rats were used, and sex differences in obesity and GERD pathophysiology (30) mean female rats should be included in future studies to enhance generalizability. Second, the 6-week follow-up period is short, precluding assessment of long-term weight stability, nutritional outcomes, and the durability of the anti-reflux effect. Third, we did not measure postoperative gut hormone levels (ghrelin, GLP-1, PYY) or gastric emptying rates, which limits our ability to mechanistically link SG-F-induced gastric partitioning to weight loss. Finally, SG-F was performed by a single surgical team, and surgical expertise may have contributed to the mild reflux seen in one SG-F rat. Standardizing the surgical technique is therefore critical for future preclinical and clinical research.

## Conclusion

In conclusion, SG-F is a safe and effective bariatric procedure for diet-induced obese rats. It yields weight loss comparable to conventional SG, simultaneously suggesting improved anti-reflux protection, and has comparable mortality and bleeding rates. By preserving gastric integrity and avoiding irreversible resection, SG-F overcomes a key clinical limitation of SG and LNS and serves as a promising alternative for obese patients with pre-existing GERD, who are currently recommended to undergo the more invasive, anatomically disruptive RYGB. These preclinical results strongly support further research, including larger animal models, long-term follow-up, and mechanistic investigations into gut hormone modulation and gastric emptying. Ultimately, well-designed pilot clinical trials are needed to translate SG-F into clinical practice, to provide a less invasive, reversible bariatric option for obese patients with GERD.

## Data Availability

The raw data supporting the conclusions of this article will be made available by the authors, without undue reservation.
